# Semi-Automated Segmentation of Bone Metastases from Whole-Body MRI: Reproducibility of Apparent Diffusion Coefficient Measurements

**DOI:** 10.3390/diagnostics11030499

**Published:** 2021-03-11

**Authors:** Alberto Colombo, Giulia Saia, Alcide A. Azzena, Alice Rossi, Fabio Zugni, Paola Pricolo, Paul E. Summers, Giulia Marvaso, Robert Grimm, Massimo Bellomi, Barbara A. Jereczek-Fossa, Anwar R. Padhani, Giuseppe Petralia

**Affiliations:** 1 Division of Radiology, IEO European Institute of Oncology IRCCS, 20141 Milan, Italy; giulia.saia@ieo.it (G.S.); fabio.zugni@ieo.it (F.Z.); paola.pricolo@ieo.it (P.P.); paul.summers@ieo.it (P.E.S.); massimo.bellomi@ieo.it (M.B.); 2 Postgraduate School in Radiodiagnostics, University of Milan, 20122 Milan, Italy; alcide.azzena@unimi.it; 3 Radiology Unit, Istituto Scientifico Romagnolo per lo Studio e la Cura dei Tumori (IRST) IRCCS, 47014 Meldola, Italy; alice.rossi@irst.emr.it; 4 Division of Radiotherapy, IEO European Institute of Oncology IRCCS, 20141 Milan, Italy; giulia.marvaso@ieo.it (G.M.); barbara.jereczek@ieo.it (B.A.J.-F.); 5 Department of Oncology and Hemato-Oncology, University of Milan, 20122 Milan, Italy; giuseppe.petralia@ieo.it; 6 MR Applications Pre-Development, Siemens Healthcare, 91052 Erlangen, Germany; robertgrimm@siemens-healthineers.com; 7 Paul Strickland Scanner Centre, Mount Vernon Cancer Centre, Northwood HA6 2RN, UK; anwar.padhani@stricklandscanner.org.uk; 8 Precision Imaging and Research Unit, Department of Medical Imaging and Radiation Sciences, IEO European Institute of Oncology IRCCS, 20141 Milan, Italy

**Keywords:** WB-MRI, DWI, ADC, quantitative analysis, bone metastases, reproducibility

## Abstract

Using semi-automated software simplifies quantitative analysis of the visible burden of disease on whole-body MRI diffusion-weighted images. To establish the intra- and inter-observer reproducibility of apparent diffusion coefficient (ADC) measures, we retrospectively analyzed data from 20 patients with bone metastases from breast (BCa; *n* = 10; aged 62.3 ± 14.8) or prostate cancer (PCa; *n* = 10; aged 67.4 ± 9.0) who had undergone examinations at two timepoints, before and after hormone-therapy. Four independent observers processed all images twice, first segmenting the entire skeleton on diffusion-weighted images, and then isolating bone metastases via ADC histogram thresholding (ADC: 650–1400 µm^2^/s). Dice Similarity, Bland-Altman method, and Intraclass Correlation Coefficient were used to assess reproducibility. Inter-observer Dice similarity was moderate (0.71) for women with BCa and poor (0.40) for men with PCa. Nonetheless, the limits of agreement of the mean ADC were just ±6% for women with BCa and ±10% for men with PCa (mean ADCs: 941 and 999 µm^2^/s, respectively). Inter-observer Intraclass Correlation Coefficients of the ADC histogram parameters were consistently greater in women with BCa than in men with PCa. While scope remains for improving consistency of the volume segmented, the observer-dependent variability measured in this study was appropriate to distinguish the clinically meaningful changes of ADC observed in patients responding to therapy, as changes of at least 25% are of interest.

## 1. Introduction

Occurring in up to 70% of patients with advanced breast cancer (BCa) or prostate cancer (PCa), bone metastases are frequently present in patients in therapy for these tumours [1]. Precise and timely assessments of therapy response in metastatic BCa and PCa are needed to ensure targeted therapies are administered efficiently [2,3]. The RECIST v1.1 criteria commonly used for evaluating response to treatment, however, are inappropriate for assessing the response of bone metastases, because bone-limited lesions are classified as “unmeasurable” [4,5]. Whole-body MRI (WB-MRI) that includes WB diffusion-weighted images (DWI) marks a paradigm shift in the assessment of treatment response of bone metastases [6,7,8]: indeed, beyond volume changes visible on conventional imaging, WB-MRI can also detect early functional changes via differences in apparent diffusion coefficient (ADC) [9,10], a quantitative index of water motility obtained from DWI [11,12]. Unlike soft tissue lesions [13], active bone lesions have higher ADC values than normal, fat-rich bone marrow [14,15], but ADC values tend to increase for both soft tissues and malignant bone lesions when there is substantial response to therapy due to an increased mobility of water molecules accompanying cell death [16,17].

As a quantitative metric, each ADC measurement is subject to uncertainty related to patient and experimental variability (physiological factors, scanner used, DWI acquisition protocol and ADC computation method) as well as to the process of drawing regions of interest from which to extract the values [18]. This last process is particularly challenging in metastatic patients when multiple lesions are distributed in the skeleton. A recent systematic analysis reported that ADC differences of at least 12% in repeated experiments could be considered true changes [19], while the inter-observer variability of mean ADC in bone metastases was about 7% and thus sufficiently low not to significantly reduce overall sensitivity to clinically relevant ADC changes [20].

The potential of WB-MRI ADC histogram analysis for monitoring of bone disease has been demonstrated but its clinical use is limited because segmentation can be influenced by observer experience and is time-consuming to perform, even with semi-automation [20,21,22,23,24]. In response to these shortcomings, a streamlined semi-automatic technique for segmenting distributed bone metastases has been developed that combines the optional calculation of heavily diffusion-weighted images [25], with thresholds over the entire image volume, manual editing, and finally, limitation of ADC values to the range of clinical interest.

The aim of this study was to determine the intra- and inter-observer reproducibility for quantitative ADC values obtained through this semi-automated approach to segmentation of bone metastases from WB-MRI by multiple observers with widely varying prior experience.

## 2. Materials and Methods

### 2.1. Population

The local ethics committee approved this retrospective single center study, and written informed consent was obtained from the subjects for use of their data. Based on a power analysis using the results of a previous study [20], 20 patients with two WB-MRI examinations were included in the study. In order to obtain a homogeneous population from the point of view of the therapy performed, patients were consecutively included if compliant with these criteria: having undergone both a baseline WB-MRI examination prior to initiating therapy and a follow-up WB-MRI examination during first-line hormone therapy following a radiological diagnosis of metastatic bone disease originating for women with invasive ductal or lobular breast carcinoma and for men with prostatic adenocarcinoma between January 2013 and March 2018. Both examinations were included in the study to represent the range of examinations occurring in clinical routine. Patients who underwent other metastases directed treatments (chemotherapy, radiotherapy, surgery) before the follow-up study were excluded.

### 2.2. WB-MRI Acquisition Protocol

The WB-MRI examinations were performed using a 1.5T MR scanner (MAGNETOM Avanto^fit^, Siemens Healthcare, Erlangen, Germany). The scanning protocol was MET-RADS-P compliant [6]. In particular, DWI scans extended from the upper border of the orbits to mid-thigh and consisted of four contiguous stations of 50 slices acquired in free-breathing using a 2D single shot echo-planar imaging (SS-EPI) sequence. Over the course of the study, two distinct shimming techniques and acquisition parameter sets (Table 1) were used for the DWI scans without changes to the *b*-values applied. Initially, a single, volumetric shim was determined for each station and applied for all slices within the station. From June 2016 onwards, slice-specific shimming was performed within each station using a prototype acquisition software provided by the machine vendor [26].

### 2.3. Image Segmentation

The WB-MRI examination images were exported in DICOM format to a reporting workstation. To allow independent evaluation of the baseline and follow-up examinations, a distinct code was applied to each examination during anonymization. Segmentation of bone metastases consisted of two sub-procedures.

**Figure 1:**
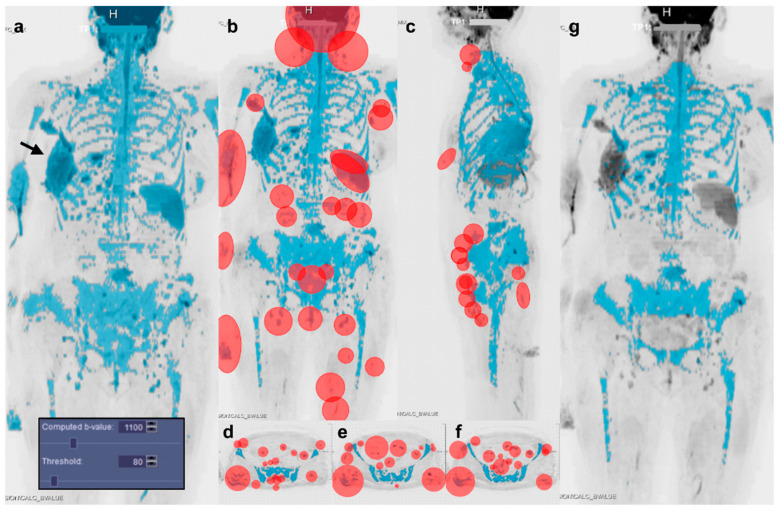
Illustration of the semi-automatic method used for bone marrow segmentation. Bone segmentation started with the observer interactively selecting a *b*-value for the calculation of a diffusion-weighted image stack (computed *b*-value) that provided good visual contrast between bone and surrounding tissues in coronal inverted gray-scale maximum intensity projection (MIP). (**a**) The observer then interactively adjusted a threshold to isolate voxels having high signal intensity on the computed *b*-value image (i.e., darker on the inverted MIP) to incorporate as much bone as possible in the resulting mask (seen overlaid in blue on the inverted MIP of the computed *b*-value DWI stack). The initial mask thus included suspected hypercellular lesions and as much bone as possible, but inevitably also included some non-bone tissues, typically brain and spinal cord, spleen, male gonads, breast implants, and sites of soft tissue inflammation or soft tissue lesions (e.g., the large soft tissue metastases along the right chest wall (arrow) and supraclavicular lymph nodes seen in this case of a 71-year-old woman with operated lobular carcinoma of the right breast, undergoing endocrine treatment). Manual editing was therefore performed to remove as much non-bone tissue as possible using a combination of: (**b**) full-depth cutting of ellipsoids (overlaid in red) positioned on the coronal MIP to eliminate brain, neck lymph nodes, soft tissues of the small pelvis, and as needed, spleen, kidneys, and lymph nodes not overlapping diseased bone, followed by (**c**) full-depth cutting of ellipsoids (overlaid in red) drawn on the sagittal MIP to eliminate soft tissues of the anterior neck, breast implants (if any), bowel, rectum, as well as inguinal and external iliac lymph nodes. If needed, (**d**,**e**,**f**) single-slice cutting of ellipsoids (overlaid in red) on individual axial, coronal or sagittal slices to eliminate soft tissues that projected over bone in the MIPs. Finally, the bone mask (**g**) was saved as a DICOM image stack.

First, bone marrow segmentation was performed using a semi-automatic approach based on signal intensity thresholding of DWI images, previously described for the direct segmentation of the visible bone metastases [22,25]. A software implementation of this method (MR Total Tumor Load, Siemens Healthcare, Erlangen, Germany) was used, that combined automatic pre-processing and computation of a volumetric ADC map (with mono-exponential fitting); observers were required to select a signal intensity threshold applied to a simulated high *b*-value image stack and manual editing to obtain a bone marrow mask (Figure 1).

**Figure 2:**
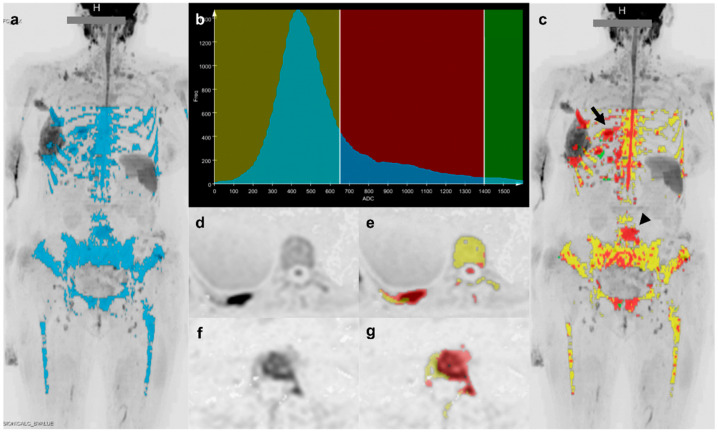
Extracting the bone metastases region of interest by applying apparent diffusion coefficient (ADC) thresholds to the bone marrow mask. Lesion segmentation started with the bone mask (**a**) seen overlaid on a coronal inverted gray-scale maximum intensity correction (MIP) being used to produce an ADC histogram (**b**) from the ADC data. The histogram was divided into three categories on the basis of two thresholds: below 650 µm^2^/s corresponding to normal bone (yellow band), between 650 µm^2^/s and 1400 µm^2^/s corresponding to lesions (red band), and above 1400 µm^2^/s corresponding to necrotic lesion or cyst (green band). The normal bone and bone lesion voxels identified in this way were then colorized as yellow and red, respectively, and overlaid on the coronal inverted gray-scale MIP (**c**) to show the localization of healthy bone and active disease. For two lesions (arrow and arrowhead in (**c**)), axial high *b*-value diffusion-weighted images and the resulting separation of bone marrow (in yellow) from metastases (in red) shows: a lesion of the posterior arc of the 10th right rib (arrow in (**c**–**e**)); a lesion of the lumbosacral spine involving transverse process and part of the vertebral body (arrowhead in (**c**,**f**,**g**)). Some residual soft tissues having ADC values in the considered range were included in the final evaluation (e.g., spinal cord in (**e**)).

Second, the bone mask and the ADC map were saved as DICOM image stacks and used in calculating ADC histograms to isolate the metastases via ad hoc functions written in Python 3.7 (Python Software Foundation, Beaverton, OR, USA). In short, a lower threshold of 650 μm^2^/s and an upper threshold of 1400 μm^2^/s were applied to the masked regions of the ADC map to remove normal bone marrow [27,28,29,30] and necrotic disease [6] (Figure 2). For the remaining voxels, which were assumed to represent bone lesions, we calculated the segmentation volume (Volume), mean (Mean_ADC), standard deviation (Std_ADC), median (Median_ADC), 5th and 95th percentiles (5%_ADC and 95%_ADC), skewness (Skewness_ADC), kurtosis (Kurtosis_ADC), and histogram entropy (Entropy_ADC) from ADC histograms. Due to signal differences between the stations obtained with the head/neck coil and the remaining body stations (acquired with anterior and posterior array coils), we limited our processing to the three body blocks covering from the upper thorax to the mid-thighs.

### 2.4. Observers

Four independent observers segmented each of the 40 DWI scans and repeated the process at least three weeks later, in a separate reading session, to minimize recall bias. None of the observers had prior experience in reporting WB-MRI. Two observers, a biomedical engineer experienced in image processing (Obs1_MASKED) and a radiologist with eight years of experience (Obs3_MASKED) had relevant background expertise in medical image processing, while the other two—a radiology resident (Obs2_MASKED) and a student radiology technologist (Obs4_MASKED)—were relatively inexperienced in image processing methods.

### 2.5. Statistical Analysis

The computed *b*-values and thresholds chosen by the observers for segmentation, and the time required to complete each segmentation were recorded. The similarity between segmentations was expressed using the Dice Similarity Coefficient (DSC) [31]. Associations between DSC and factors potentially influencing segmentation similarity were assessed using factorial ANOVA and Spearman’s coefficient (ρ_s_) for categorical and continuous variables, respectively. The factors considered were patient sex, age, treatment status at the time of WB-MRI (baseline or follow-up examination), number of MET-RADS-P skeletal regions with metastases, and shimming technique [6]. In light of a strong effect of sex, subsequent analyses were performed separately for men with PCa and women with BCa, and the Mann-Whitney test was used to compare measures.

The distribution of Mean_ADC and of the other histogram parameter values was expressed as their average across readers. Measures of the second reading session were considered in order to minimize the learning curve effect for the first segmentation.

The Bland-Altman method [32,33] was used to evaluate intra-observer (comparing the first and second segmentations for each observer), and inter-observer (comparing pairs of readers within each of the two reading sessions) reproducibility of Mean_ADC. Dependence of absolute differences on the mean of measurements was assessed using Kendall’s tau (τ_b_), and the mean intra- and inter-observer bias and 95% limits of agreement were determined. The correlation between the volume of segmentation and the variability of Mean_ADC (mean difference among intra-observer and inter-observer measurements) was evaluated with Spearman’s correlation coefficient.

Intra- and inter-observer reproducibility of the other ADC histogram statistics were measured using Intraclass Correlation Coefficients (ICC) with 95% confidence intervals, calculating absolute concordance using a two-way model with mixed effects and single measurements [34]. For both DSC and ICC, the following classification scale was used to evaluate similarity/reproducibility: poor (DSC/ICC < 0.50), modest (0.50 ≤ DSC/ICC < 0.75), good (0.75 ≤ DSC/ICC < 0.90), and excellent (DSC/ICC ≥ 0.90). We considered results of *p* < 0.05 significant and analyses were performed with the R software package (R 2018, version 3.5.1, Vienna, Austria).

## 3. Results

### 3.1. Population

**Figure 3:**
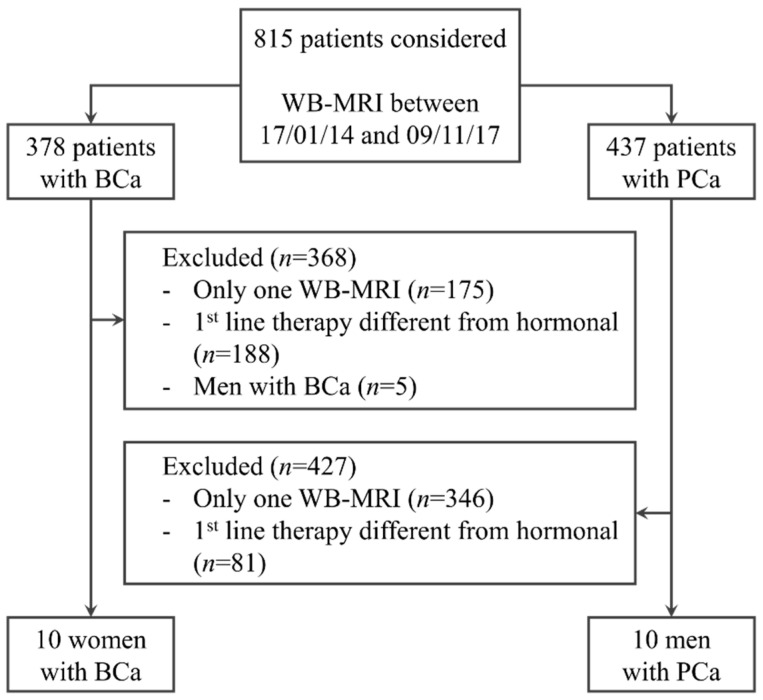
Diagram of the patient selection workflow. Of the 815 patients who had undergone whole-body MRI (WB-MRI) in our institution during the study period, 20 patients, 10 men with prostate cancer (PCa) and 10 women with breast cancer (BCa), satisfied the inclusion criteria of having undergone more than one WB-MRI, and being on first-line hormonal therapy.

Of the 378 BCa and 437 PCa patients who underwent WB-MRI in the study period (Figure 3), 10 women with BCa and 10 men with PCa met the inclusion criteria. Clinical and demographic characteristics of the patients are summarized in Table 2.

Follow-up examinations were performed an average 206 days after the baseline examination (range: 90–373 days). Of the 40 WB-MRI examinations analyzed, 23 were acquired using station-based shimming (15 BCa and 8 PCa patients), and 17 with slice-specific shimming (5 BCa and 12 PCa patients).

### 3.2. Segmentation Settings and Duration

The time between the first and the second reading sessions ranged from three to four weeks across the four observers. A summary of the settings used, and times required for evaluation is given in Table S1 (Supplementary Material). Between the observers, the average *b*-values used for the computed *b*-value image ranged from 994 ± 23 to 1057 ± 67 s/mm^2^, with an overall mean of 1012 s/mm^2^. The threshold signal intensity for initial segmentation ranged from 32.5 ± 20.5 to 47.3 ± 41.6 with an overall mean of 41.

The time required to perform a segmentation ranged from 4 to 38 min. For the experienced observers (Obs 1, Obs 3), the average segmentation time across all examinations was about 11 min shorter (12 vs. 23 min) and the range of times for individual patients narrower (4 to 28 min vs. 8 to 38 min) than for the inexperienced observers (Obs 2, Obs 4). On average, the observers were 2.1 ± 0.4 min faster in the second segmentation session.

### 3.3. Factors Influencing Segmentation Similarity

Patient sex was significantly associated with mean intra-observer DSC values (*p* < 0.0001), which were greater for women with BCa (Figure S1, Supplementary Material). A smaller, but still significant association was also seen with respect to the shimming technique used (*p* < 0.01), with station-based shimming tending to yield a higher DSC. Treatment (baseline vs. follow-up examination) had no effect (*p* = 0.81). A moderate positive correlation between DSC and number of skeletal regions with metastases (ρ_s_ = 0.58, *p* < 0.0001) was also noted.

### 3.4. Distribution of Quantitative Parameters Values

In women with BCa, the average Mean_ADC measurement of the four observers was 936.6 ± 101.9 μm^2^/s at baseline, and 945.4 ± 91.3 μm^2^/s at follow-up WB-MRI. Similar values were found in men with PCa, for whom Mean_ADC was 963.5 ± 91.5 μm^2^/s and 1033.5 ± 84.1 μm^2^/s at baseline and follow-up, respectively. The Table S2 (Supplementary Material) shows the distribution of average values, at baseline and follow-up, for the other quantitative histogram parameters.

### 3.5. Intra- and Inter-Observer Reproducibility Analysis

Overall, the mean intra-observer DSC value was modest (0.67) but was significantly higher in women with BCa than in men with PCa (good: 0.78 vs. modest: 0.55, *p* < 0.0001).

For women with BCa, the intra-observer Bland-Altman bias and limits of agreement of Mean_ADC were 0.5% (−5.2%, 6.0%) for an average measure of 942.9 μm^2^/s, and for men with PCa, they were 0.5% (−9.0%, 9.9%) and 1000.8 μm^2^/s. No significant correlation was found between the volume of lesion segmented and the variability of Mean_ADC in women with BCa (ρ_s_ = −0.35, *p* = 0.13), or in men with PCa (ρ_s_ = 0.15, *p* = 0.50). In women with BCa, the intra-observer ICC for Mean_ADC showed excellent agreement (95% CI, 0.90–0.98), while in men with PCa, it was modest to excellent (95% CI, 0.63–0.92). Across the parameters considered, the intra-observer ICCs tended to be greater, and the 95% confidence intervals narrower, for women with BCa than for men with PCa.

Results of the intra- and inter-observer reproducibility analyses are summarized in Table 3. Detailed information regarding DSC, Bland Altman bias and limits of agreement and ICC are reported, respectively, in Tables S3–S5 (Supplementary Material). Neither intra- nor inter-observer differences in ADC showed relevant dependence on mean ADC (τ_b_ = 0.12 with *p* = 0.27 and τ_b_ = −0.34 with *p* < 0.01).

**Figure 4:**
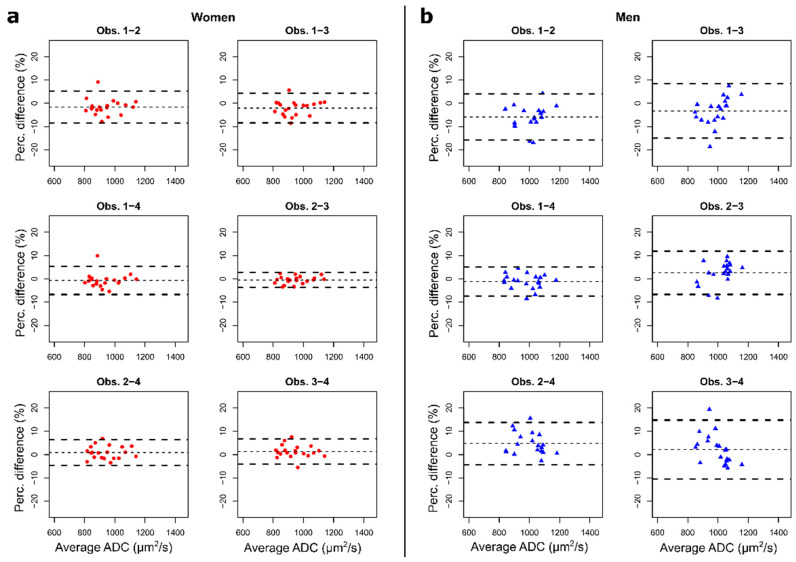
Bland-Altman plots of inter-observer mean apparent diffusion coefficient (ADC) measures of bone lesions. Each plot represents the percentage difference between the measures of a pair of observers compared to the average of their measures in the second reading session. In our cohort, (**a**) excellent reproducibility was observed in women with breast cancer, with bias and 95% limits of agreement below ±2.5% and ±8.5%, respectively. (**b**) Higher variability was observed in men with prostate cancer, with bias and 95% limits of agreement below ±6% and ±16%, respectively.

Overall, the mean inter-observer DSC showed modest segmentation similarity (0.52 and 0.55 for first and second reading respectively). The DSC values were significantly higher in women with BCa (0.67 for the first reading and 0.71 for the second) than in men with PCa (*p* < 0.0001), where poor similarity was observed (0.37 and 0.40). Inter-observer bias and limits of agreement of Mean_ADC in the second reading session for women with BCa and men with PCa were, respectively, 1.2% (−6.0%, 5.1%) and 3.3% (−9.9%, 9.6%), for average measures of 941.0 μm^2^/s and 998.5 μm^2^/s (Figure 4).

**Figure 5:**
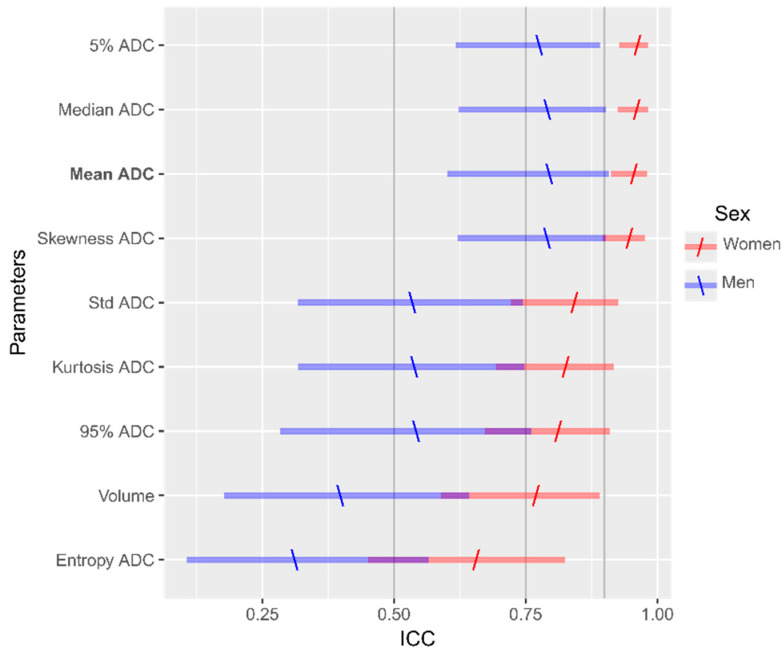
Graphical representation of the inter-observer Intraclass Correlation Coefficients (ICC) with lower and upper limits of the 95% confidence intervals calculated for parameters derived from the apparent diffusion coefficient histogram. The population is divided by sex (blue: men with prostate cancer, red: women with breast cancer), back-slashes and forward slashes represent the estimated ICC values for men and women, respectively. For our cohort, the intervals were narrower and ICC values nearer to 1 in women, indicating greater reproducibility than in men.

In the inter-observer analysis, the lesion volume and variability of Mean_ADC were not correlated: though a weak negative trend was observed in women with BCa (ρ_s_ =−0.42, *p* = 0.07), it was not seen in men with PCa (ρ_s_ = −0.16, *p* = 0.49). The inter-observer ICC for Mean_ADC showed excellent reproducibility in women with BCa (95% CI, 0.91–0.98), as opposed to the modest to excellent reproducibility obtained in men with PCa (95% CI, 0.60–0.91). The inter-observer ICC analysis of the other histogram statistics showed greater reproducibility and narrower 95% confidence intervals, for women with BCa than for men with PCa (Figure 5).

## 4. Discussion

Prior studies have demonstrated the ability of WB-MRI-based ADC measurements to monitor treatment response in patients with metastatic bone disease [20]. However, available approaches to segmentation of bone metastases are dependent on radiological expertise and are too time consuming for realistic clinical use [22]. As a precursor to the use of WB-MRI in the monitoring of treatment in metastatic disease, we examined the intra- and inter-observer reproducibility of metastatic bone lesion segmentation and of the corresponding ADC values obtained using a semi-automated tool for segmenting dispersed skeletal lesions, by observers with diverse clinical expertise.

Our process starts with segmentation of bone, for which the *b*-values chosen to provide optimal contrast between bone marrow and soft tissues on the calculated diffusion weighted image were close to 1000 s/mm^2^ across the cohort of patients. Blackledge et al. [25] found similar *b*-values yielded simulated images (median: 1070 s/mm^2^, range: 715–1660 s/mm^2^) that were optimal for direct lesion segmentation. Both results point to the optimal *b*-value for segmentation being different from that recommended for acquisition in the MET-RADS-P and MY-RADS guidelines (800 s/mm^2^) where scan time and contrast to noise must be accommodated [6,35]. This is not a significant obstacle as the calculation of a higher *b*-value image for use in segmentation can readily be obtained via a mono-exponential calculation.

The second step in the segmentation process involved the selection of a threshold based on the calculated *b*-value image. Due to a lack of standardization of the MRI signal intensities, this threshold is likely to depend on acquisition settings, field-strength, and system hardware. Normalization of the DWI signal intensities by the muscle signal intensity has been proposed as a strategy for threshold selection that is independent of the diffusion MRI acquisition settings (e.g., gain settings, b-value gradient, coil and fat-suppression method) [27].

The two more experienced observers took an average of 12 min (range 4–28 min) to complete each segmentation, while the less experienced observers averaged 23 min. These times compare favourably with the roughly 30 min reported by Blackledge et al. [22], for segmentation of metastases by experienced observers. Achieving segmentation in a clinically acceptable time is a key obstacle to be overcome for the use of quantitative WB-MRI in monitoring treatment response in bone metastases.

In assessing factors that influence segmentation similarity, as indicated by the mean intra-observer DSC values, we found that patient sex had a particularly strong effect, with higher DSC values being seen in women with BCa than in men with PCa (0.78 vs. 0.55). We attribute this difference to hyperintensity of bone marrow on diffusion-weighted images in women, a feature observed in previous studies [36,37]. If the marrow is hyperintense, the threshold applied to the high *b*-value images allows a cleaner separation from other tissues, and thus requires less manual editing. This makes semi-automatic segmentation of bone marrow particularly suitable for women, while additional post-processing would be required in men to achieve matching levels of segmentation similarity [38]. Shimming technique had a small but significant effect on DSC values. This likely relates to different signal-to-noise ratios in the diffusion-weighted images due to the difference in shim quality, but has not been found to result in significant differences in ADC values [26,39]. We have therefore incorporated data obtained using both shim techniques in this study to evaluate the variability related to the observers performing the analysis on each image independently.

Taking the inter-observer 95% limits of agreement of Mean_ADC in bone metastases as representative of observer performance, changes of 6% in women with BCa and 10% in men with PCa could be considered beyond the observer-related variability. These values are similar to the 7% reported by Blackledge et al. [20], who used a more time-consuming approach to segmenting bone metastases. These differences suggest that, with this method, a better sensitivity to ADC change in metastases can be expected for women with BCa than for men with PCa.

On top of the observer-related variability documented here, test-retest experimental variability needs to be considered to establish the magnitude of change in ADC that must occur before it can be unequivocally detected. Winfield et al. reported that mean ADC increases of >12% could be considered real changes in repeated experiments [19]. It is reasonable therefore, to expect that the test-retest variability of WB-MRI for ADC of bone metastases will be clinically acceptable as the MET-RADS-P and MY-RADS guidelines indicate that increases in Mean_ADC values induced by therapy should be at least 25% between baseline and follow-up in case of “likely” response, and at least 40% in case of “very likely” response [6,35].

Blackledge et al. [20] reported excellent reproducibility not only for mean ADC, but also for metastatic volume parameters, consistent with the results of Perez-Lopez et al. [23]. While our results were similar for reproducibility of mean and median ADC, reproducibility of the segmentation volume was lower, yielding poor-modest DSC values. The variability of the lesion volumes and of the DSC values on the other hand, likely reflects the remaining subjectivity in the initial segmentation and in the manual elimination of residual soft tissues. This variability limits the clinical applicability of volume-related measures obtained with this method, particularly for evaluating men with PCa. Future studies should seek to reduce the variability of volume among readers while improving the repeatability of disease segmentation. The relative immunity of the ADC values in the face of variable lesion volume, suggests that the use of the thresholds for isolating the metastases imposes a degree of robustness in terms of ADC values, making this parameter an interesting complementary tool to radiological evaluation.

The small sample size and single center nature of our study are limitations that may restrict generalizability to our work. In addition, there was no ground truth for the segmentation of metastases (e.g., manual segmentation performed by a radiologist experienced in WB-MRI), and consequently we cannot comment on segmentation accuracy. Furthermore, we have addressed only the issues of intra and inter-observer reproducibility. Having found robust ADC value extraction, it is reasonable to pursue a test-retest study to establish the magnitude of change that can be detected with confidence. Finally, two different shimming techniques (station- and slice-specific) were used during the observation period: other studies have found the difference in ADC values between these techniques to be small, but their inclusion may have inflated the observer-related variability.

## 5. Conclusions

While scope remains for improving the consistency of the volume of bone metastases segmented, the segmentation method evaluated in this study demonstrates good to excellent levels of intra- and inter-observer reproducibility in measuring mean ADC, particularly for women with BCa. Noting that, according to MET-RADS-P and MY-RADS guidelines, the cut-off for clinically meaningful changes in mean ADC in patients who respond to therapy is at least 25%, the observer-dependent variability with the proposed approach is acceptable. Although observer-dependent variability was greater in men with PCa, the technique is likely to still be adequate for detecting responses to therapy at higher mean ADC change thresholds.

## Figures and Tables

**Table 1:** Whole-body diffusion-weighted image acquisition protocols.

	Station-Specific Shim	Slice-Specific Shim
Sequence	Diffusion-Weighted SS-EPI	Diffusion-Weighted SS-EPI
Orientation	Transversal	Transversal
*b*-value (s/mm^2^)	50, 900	50, 900
Encoding mode	3-scan trace	3D-diagonal
Averages per *b*-value	6, 6	5, 15
Repetition Time (ms)	9000	6550
Echo Time (ms)	67	62
Fat Saturation	STIR	STIR
Inversion Time (ms)	180	180
Field of View (mm)	337 × 450	390 × 429
Slice thickness (mm)	5.0	5.0
Gap between slices (mm)	0.0	0.0
Voxel size (mm^3^)	1.8 × 1.8 × 5.0	1.6 × 1.6 × 5.0
Acquisition time (min)	22:00	15:02

SS-EPI = Single-Shot Echo-Planar Imaging, STIR = Short Tau Inversion Recovery.

**Table 2:** A summary of clinical and demographical information.

		Men with PCa	Women with BCa
Patients	Number	10	10
	Age at baseline (years) ^1^	67.4 (51–77)	62.3 (31–76)
	No. of skeletal regions with metastasis ^1^	3.7 (2–6)	5.0 (3–6)
Type of primary tumour	Prostatic adenocarcinoma	10	0
	Invasive ductal breast cancer	0	3
	Invasive lobular breast cancer	0	7
No. of patients with metastasis for each skeletal region	Skull	0	2
	Spine (cervical)	3	9
	Spine (thorax)	6	7
	Spine (lumbosacral)	8	9
	Thorax	7	8
	Pelvis	9	10
	Limbs	4	7
Other sites of disease	Lymph nodes	60% (6/10)	50% (5/10)
	Visceral	0% (0/10)	40% (4/10)
	Local disease	50% (5/10)	10% (1/10)
	Other (muscles)	0% (0/10)	10% (1/10)
WB-MRI examinations	Observation period	17/01/14–29/05/17	03/09/15–09/11/17
	No. baseline/follow-up	10/10	10/10
	Days between baseline and follow-up ^1^	213.8 (90–373)	197.9 (109–291)
	No. with station-/slice-specific shim	8/12	15/5

WB-MRI = Whole-Body MRI, PCa = Prostate cancer, BCa = Breast cancer, ^1^ Mean (range).

**Table 3:** Summary of observer-dependent reproducibility metrics.

		Women with BCa (*n* = 20)	Men with PCa (*n* = 20)
**Intra-observer**			
DSC	mean ± std dev	0.78 ± 0.14	0.55 ± 0.21
BA (Mean_ADC)	bias (LoA)	0.5% (−5.2%, 6.0%)	0.5% (−9.0%, 9.9%)
ICC (Mean_ADC)	estimate (95% CI)	0.96 (0.90–0.98)	0.82 (0.63–0.92)
**Inter-observer** (**2nd reading**)			
DSC	mean ± std dev	0.71 ± 0.16	0.40 ± 0.19
BA (Mean_ADC)	bias (LoA)	1.2% (−6.0%, 5.1%)	3.3% (−9.9%, 9.6%)
ICC (Mean_ADC)	estimate (95% CI)	0.96 (0.91–0.98)	0.79 (0.60–0.91)

BCa = breast cancer, PCa = prostate cancer, DSC = Dice Similarity Coefficient, BA = Bland-Altman, LoA = Limits of Agreement, ICC = Intraclass Correlation Coefficients, Mean_ADC = mean of apparent diffusion coefficient distribution.

## Data Availability

The data is available for review from the corresponding author on request.

## Supplementary Materials

The following are available online at https://www.mdpi.com/2075-4418/11/3/499/s1, Figure S1: Effect of sex, treatment status, and shimming technique on Dice Similarity Coefficients (DSC), Table S1: Segmentation settings and duration, Table S2: Distribution descriptors of parameters measured in the second reading, Table S3: Summary tables of intra-observer (on-diagonal) and inter-observer (off-diagonal) mean Dice Similarity Coefficients (DSC) by reading, Table S4: Summary of intra- and inter- observer Bland-Altman analysis results (bias and limits of agreement) for mean apparent diffusion coefficient (ADC) as percentages of the mean of the measures, Table S5 Intra- and inter-observer Intra-class Correlation Coefficients (ICC).
