# Supplementary material for: Semi-Automated Segmentation of Bone Metastases from Whole-Body MRI: Reproducibility of Apparent Diffusion Coefficient Measurements
Source: Diagnostics (Basel). 2021 Mar 11;11(3):499. doi: 10.3390/diagnostics11030499 (PMC7998160; doi:10.3390/diagnostics11030499)
Supplement: Supplementary file 1 [file diagnostics-11-00499-s001.pdf]

diagnostics

Article

Semi-automated Segmentation of Bone Metastases from Whole-Body MRI: Reproducibility of Apparent Diffusion Coefficient Measurements

Supplementary Material

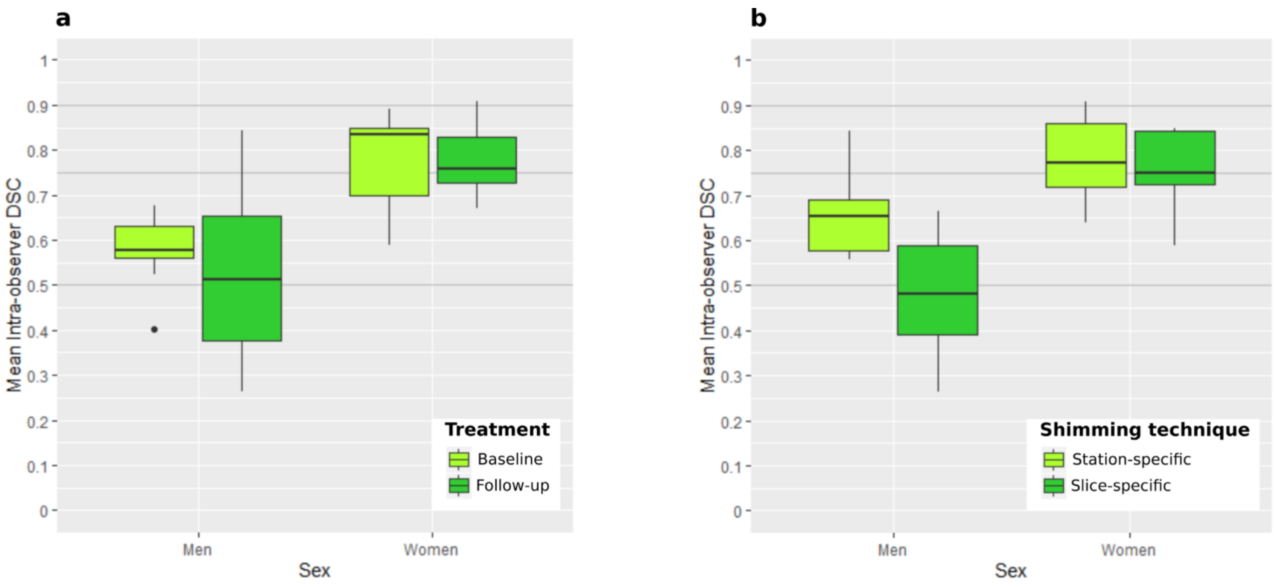

Figure S1. Effect of sex, treatment status and shimming technique on Dice Similarity Coefficients (DSC). The box-plots show the distribution of mean intra-observer Dice Similarity Coefficients (DSC), dividing the population by patient sex, treatment status (baseline or follow-up) and shimming technique used (station-specific or slice-specific). A significant difference in DSC was apparent between men and women (p=0.0001). (a) The difference between DSC measured in baseline and follow-up whole-body MRI examinations wasn´t significant for men (p=0.63) or women (p=0.97). (b) While shimming technique had minimal effect on DSC values in women (p=0.54), a significant difference was observed in men (p=0.02).

diagnostics

Table S1. Segmentation settings and duration

Observer, reading session	Comp_b (s/mm^2)	Threshold (SI)	Duration (min)
**Obs 1, 1st**	1057.5 ± 67.5	42.0 ± 40.1	13.9 ± 5.7
**Obs 1, 2nd**	1057.5 ± 59.4	36.2 ± 23.7	13.3 ± 6.1
**Obs 2, 1st**	995.0 ± 38.9	45.1 ± 27.3	22.1 ± 7.8
**Obs 2, 2nd**	993.8 ± 23.2	47.3 ± 41.6	17.8 ± 8.1
**Obs 3, 1st**	986.3 ± 83.2	44.7 ± 35.1	12.2 ± 5.8
**Obs 3, 2nd**	988.8 ± 50.0	45.0 ± 39.6	9.4 ± 5.0
**Obs 4, 1st**	1011.3 ± 53.7	32.5 ± 20.5	27.9 ± 6.5
**Obs 4, 2nd**	1007.5 ± 26.7	33.0 ± 20.0	25.3 ± 7.6

Comp_b = simulated diffusion-weighted image stack, SI = Signal Intensity.

diagnostics

Table S2. Distribution descriptors of parameters measured in the second reading

		Patients with BCa		Patients with PCa	
	**Unit**	**Baseline**	**Follow-up**	**Baseline**	**Follow-up**
**Mean_ADC**	$\mu m^2/s$	936.6 ± 101.9	945.4 ± 91.3	963.5 ± 91.5	1033.5 ± 84.1
**Median_ADC**	$\mu m^2/s$	899.0 ± 143.2	913.0 ± 129.6	945.0 ± 132	1039.5 ± 119.0
**5%_ADC**	$\mu m^2/s$	686.0 ± 59.8	669.0 ± 28.3	675.5 ± 27.2	708.0 ± 57.4
**95%_ADC**	$\mu m^2/s$	1302.0 ± 42.4	1319.0 ± 41.5	1324.5 ± 46.4	1343.9 ± 23.6
**Std_ADC**	$\mu m^2/s$	193.2 ± 24.7	206.5 ± 15.6	207.2 ± 21.1	199.6 ± 22.2
**Skewness_ADC**	-	0.5 ± 0.7	0.5 ± 0.6	0.4 ± 0.6	-0.1 ± 0.6
**Kurtosis_ADC**	-	-0.2 ± 0.8	-0.6 ± 0.8	-0.6 ± 0.9	-0.7 ± 0.6
**Entropy_ADC**	-	10.0 ± 0.7	9.7 ± 0.6	9.3 ± 0.7	9.0 ± 0.7
**Volume**	cm^3	429.2 ± 297.5	281.2 ± 165.2	206.9 ± 159.1	143.2 ± 118.6

BCa = Breast cancer, PCa = Prostate cancer.

diagnostics

MDPI

Table S3. Summary tables of intra-observer (on-diagonal) and inter-observer (off-diagonal) mean Dice Similarity Coefficients (DSC) by reading

Entire Cohort

Observer	1	2	3	4
**1**	0.69 ± 0.21	0.48 ± 0.27	0.60 ± 0.26	0.57 ± 0.21
**2**	0.52 ± 0.27	0.59 ± 0.23	0.52 ± 0.21	0.43 ± 0.25
**3**	0.57 ± 0.23	0.57 ± 0.21	0.66 ± 0.22	0.52 ± 0.20
**4**	0.64 ± 0.22	0.50 ± 0.25	0.51 ± 0.24	0.71 ± 0.19

Women

Observer	1	2	3	4
**1**	0.80 ± 0.13	0.70 ± 0.14	0.78 ± 0.16	0.67 ± 0.17
**2**	0.72 ± 0.17	0.73 ± 0.16	0.64 ± 0.16	0.58 ± 0.17
**3**	0.73 ± 0.13	0.73 ± 0.14	0.81 ± 0.12	0.63 ± 0.15
**4**	0.75 ± 0.17	0.64 ± 0.19	0.68 ± 0.18	0.78 ± 0.17

Men

Observer	1	2	3	4
**1**	0.59 ± 0.22	0.27 ± 0.18	0.42 ± 0.21	0.48 ± 0.21
**2**	0.32 ± 0.20	0.46 ± 0.22	0.40 ± 0.17	0.29 ± 0.23
**3**	0.41 ± 0.20	0.42 ± 0.15	0.52 ± 0.19	0.40 ± 0.19
**4**	0.53 ± 0.22	0.36 ± 0.23	0.34 ± 0.16	0.65 ± 0.20

Legend:

Intra-observer DSC
Inter-observer DSC (1st reading)
Inter-observer DSC (2nd reading)

diagnostics

Table S4. Summary of intra- and inter- observer Bland-Altman analysis results (bias and limits of agreement) for mean apparent diffusion coefficient (ADC) as percentages of the mean of the measures

Intra-observer analysis

Observers	1	2	3	4
**Overall**	0.5 (-6.5 - 7.5); 954.1	0.8 (-11.6 - 13.2); 992.9	0.2 (-7.3 - 7.8); 978.9	0.1 (-4.6 - 4.9); 961.4
**Women**	-0.2 (-5.3 - 4.9); 929.8	1.2 (-7.4 - 9.7); 951.3	0.4 (-3.2 – 4.0); 952.2	0.2 (-4.9 - 5.3); 938.2
**Men**	1.1 (-7.2 - 9.4); 978.3	0.5 (-14.6 - 15.7); 1034.5	0.1 (-9.8 – 10.0); 1005.6	0.1 (-4.4 - 4.6); 984.6

Inter-observer analysis (1st reading)

Observers:	1-2	1-3	1-4	2-3	2-4	3-4
**Overall**	-4.2 (-17.4 – 9.0); 976.7	-2.5 (-9.9 – 5.0); 968.2	-0.6 (-7.8 - 6.7); 959.2	1.7 (-10.8 - 14.2); 988.5	3.6 (-9.9 - 17.0); 979.5	1.9 (-6.0 - 9.7); 971
**Women**	-2.9 (-12.9 - 7); 942.9	-2.7 (-7.8 - 2.4); 941.5	-1.1 (-7.4 - 5.2); 934	0.3 (-8.5 – 9.0); 955.4	1.9 (-8.6 - 12.4); 947.9	1.6 (-3.9 - 7.1); 946.5
**Men**	-5.3 (-20.7 - 10.0); 1010.5	-2.2 (-11.4 - 6.9); 994.8	-0.1 (-8.2 - 7.9); 984.4	3.1 (-11.7 - 17.8); 1021.7	5.2 (-10.0 - 20.3); 1011.2	2.1 (-7.5 - 11.7); 995.6

Inter-observer analysis (2nd reading)

Observers:	1-2	1-3	1-4	2-3	2-4	3-4
**Overall**	-3.8 (-13.4 - 5.8); 970.3	-2.7 (-12.2 - 6.8); 964.8	-0.9 (-7.0 - 5.1); 656.2	1.1 (-6.6 - 8.8); 983.2	2.9 (-5.6 - 11.4); 974.7	1.8 (-8.0 - 11.6); 969.2
**Women**	-1.6 (-8.4 - 5.2); 938.2	-2.1 (-8.4 - 4.2); 940.5	-0.7 (-6.7 - 5.3); 934	-0.5 (-3.7 - 2.8); 948	0.9 (-4.6 - 6.4); 941.6	1.4 (-4.1 - 6.8); 943.8
**Men**	-5.9 (-15.7 – 4.0); 1002.3	-3.3 (-15.0 - 8.4); 989.1	-1.1 (-7.3 - 5.1); 978.5	2.6 (-6.7 - 11.9); 1018.5	4.7 (-4.3 - 13.8); 1007.9	2.1 (-10.5 - 14.7); 994.6

Note. - Values are: Bias (Lower – Upper 95% limits of agreement) mean of the underlying measurements.

Bias and limits of agreement are expressed as percentages of the mean underlying measurements.

diagnostics

MDPI

Table S5. Intra- and inter-observer Intra-class Correlation Coefficients (ICC)

		Intra-observer					*Inter-observer*	
		**1**	**2**	**3**	**4**	**Mean (intra-)**	**1st reading**	**2nd reading**
**Mean ADC**	Overall	0.94 (0.89 - 0.97)	0.81 (0.67 - 0.90)	0.92 (0.85 - 0.95)	0.97 (0.95 - 0.99)	0.91 (0.84 - 0.95)	0.83 (0.73 - 0.90)	0.88 (0.80 - 0.94)
	Women	0.97 (0.93 - 0.99)	0.91 (0.79 - 0.96)	0.98 (0.96 - 0.99)	0.97 (0.92 - 0.99)	0.96 (0.90 - 0.98)	0.91 (0.82 - 0.96)	0.96 (0.91 - 0.98)
	Men	0.9 (0.77 - 0.96)	0.61 (0.23 - 0.83)	0.81 (0.58 - 0.92)	0.98 (0.95 - 0.99)	0.82 (0.63 - 0.92)	0.72 (0.53 - 0.86)	0.79 (0.60 - 0.91)
**Median ADC**	Overall	0.93 (0.87 - 0.96)	0.77 (0.60 - 0.87)	0.91 (0.84 - 0.95)	0.97 (0.95 - 0.99)	0.90 (0.82 - 0.94)	0.82 (0.73 - 0.89)	0.89 (0.82 - 0.93)
	Women	0.97 (0.94 - 0.99)	0.86 (0.68 - 0.94)	0.98 (0.96 - 0.99)	0.97 (0.92 - 0.99)	0.95 (0.87 - 0.98)	0.89 (0.80 - 0.95)	0.96 (0.93 - 0.98)
	Men	0.88 (0.72 - 0.95)	0.58 (0.19 - 0.81)	0.80 (0.55 - 0.91)	0.98 (0.94 - 0.99)	0.81 (0.60 - 0.92)	0.72 (0.54 - 0.86)	0.79 (0.62 - 0.90)
**5% ADC**	Overall	0.88 (0.78 - 0.93)	0.94 (0.90 - 0.97)	0.81 (0.67 - 0.89)	0.97 (0.94 - 0.98)	0.90 (0.82 - 0.94)	0.88 (0.81 - 0.93)	0.87 (0.80 - 0.92)
	Women	1.00 (0.99 - 1.00)	0.98 (0.94 - 0.99)	0.99 (0.97 - 1.00)	0.98 (0.95 - 0.99)	0.98 (0.96 - 0.99)	0.95 (0.90 - 0.98)	0.96 (0.93 - 0.98)
	Men	0.73 (0.43 - 0.88)	0.91 (0.78 - 0.96)	0.65 (0.32 - 0.84)	0.96 (0.90 - 0.98)	0.81 (0.61 - 0.92)	0.81 (0.66 - 0.91)	0.78 (0.62 - 0.89)
**95% ADC**	Overall	0.92 (0.85 - 0.96)	0.81 (0.67 - 0.90)	0.87 (0.76 - 0.93)	0.92 (0.86 - 0.96)	0.88 (0.78 - 0.93)	0.66 (0.50 - 0.78)	0.71 (0.56 - 0.82)
	Women	0.95 (0.88 - 0.98)	0.89 (0.75 - 0.95)	0.90 (0.76 - 0.96)	0.90 (0.77 - 0.96)	0.91 (0.79 - 0.96)	0.76 (0.59 - 0.89)	0.81 (0.67 - 0.91)
	Men	0.86 (0.68 - 0.94)	0.05 (-0.42 - 0.48)	0.74 (0.45 - 0.89)	0.94 (0.85 - 0.98)	0.65 (0.39 - 0.82)	0.42 (0.20 - 0.66)	0.54 (0.28 - 0.76)
**Std ADC**	Overall	0.87 (0.77 - 0.93)	0.85 (0.74 - 0.92)	0.83 (0.70 - 0.91)	0.93 (0.88 - 0.96)	0.87 (0.77 - 0.93)	0.66 (0.52 - 0.78)	0.68 (0.55 - 0.8)
	Women	0.94 (0.85 - 0.97)	0.94 (0.85 - 0.98)	0.96 (0.90 - 0.98)	0.90 (0.78 - 0.96)	0.94 (0.85 - 0.97)	0.84 (0.71 - 0.92)	0.84 (0.72 - 0.93)
	Men	0.81 (0.58 - 0.92)	0.75 (0.46 - 0.89)	0.71 (0.39 - 0.87)	0.96 (0.90 - 0.98)	0.81 (0.59 - 0.92)	0.47 (0.25 - 0.70)	0.53 (0.32 - 0.74)
**Skewness ADC**	Overall	0.95 (0.90 - 0.97)	0.76 (0.59 - 0.87)	0.90 (0.82 - 0.95)	0.97 (0.95 - 0.99)	0.9 (0.82 - 0.94)	0.79 (0.68 - 0.87)	0.88 (0.80 - 0.93)
	Women	0.97 (0.92 - 0.99)	0.89 (0.76 - 0.96)	0.98 (0.95 - 0.99)	0.96 (0.89 - 0.98)	0.95 (0.88 - 0.98)	0.88 (0.77 - 0.94)	0.95 (0.90 - 0.98)
	Men	0.92 (0.81 - 0.97)	0.51 (0.10 - 0.77)	0.78 (0.53 - 0.91)	0.98 (0.96 - 0.99)	0.80 (0.6 - 0.91)	0.68 (0.48 - 0.84)	0.79 (0.62 - 0.90)
**Kurtosis ADC**	Overall	0.90 (0.83 - 0.95)	0.75 (0.58 - 0.86)	0.76 (0.59 - 0.87)	0.92 (0.86 - 0.96)	0.83 (0.71 - 0.91)	0.54 (0.38 - 0.69)	0.69 (0.55 - 0.81)
	Women	0.91 (0.79 - 0.96)	0.88 (0.73 - 0.95)	0.91 (0.78 - 0.96)	0.88 (0.72 - 0.95)	0.89 (0.76 - 0.96)	0.67 (0.47 - 0.83)	0.83 (0.69 - 0.92)
	Men	0.89 (0.74 - 0.96)	0.53 (0.12 - 0.78)	0.61 (0.26 - 0.82)	0.97 (0.92 - 0.99)	0.75 (0.51 - 0.89)	0.39 (0.17 - 0.64)	0.54 (0.32 - 0.75)
**Entropy ADC**	Overall	0.71 (0.51 - 0.84)	0.37 (0.09 - 0.61)	0.67 (0.45 - 0.81)	0.67 (0.46 - 0.81)	0.60 (0.38 - 0.76)	0.36 (0.19 - 0.55)	0.57 (0.40 - 0.72)
	Women	0.81 (0.58 - 0.92)	0.70 (0.38 - 0.87)	0.88 (0.72 - 0.95)	0.72 (0.42 - 0.88)	0.78 (0.53 - 0.90)	0.54 (0.30 - 0.75)	0.66 (0.45 - 0.82)
	Men	0.62 (0.27 - 0.83)	-0.06 (-0.34 - 0.31)	0.20 (-0.17 - 0.56)	0.51 (0.11 - 0.77)	0.32 (-0.03 - 0.62)	0.12 (-0.02 - 0.35)	0.31 (0.11 - 0.57)
**Volume**	Overall	0.79 (0.64 - 0.88)	0.69 (0.48 - 0.82)	0.84 (0.73 - 0.91)	0.77 (0.61 - 0.87)	0.77 (0.61 - 0.87)	0.59 (0.39 - 0.75)	0.72 (0.58 - 0.83)
	Women	0.90 (0.78 - 0.96)	0.82 (0.59 - 0.92)	0.92 (0.79 - 0.97)	0.74 (0.45 - 0.89)	0.84 (0.65 - 0.94)	0.68 (0.43 - 0.85)	0.77 (0.59 - 0.89)
	Men	0.60 (0.24 - 0.82)	-0.05 (-0.34 - 0.33)	0.20 (-0.16 - 0.55)	0.72 (0.43 - 0.88)	0.37 (0.04 - 0.64)	0.27 (0.07 - 0.52)	0.40 (0.18 - 0.64)

Note. - ADC = Apparent Diffusion Coefficient, Std = Standard deviation.
